# H2AX phosphorylation and DNA damage kinase activity are dispensable for herpes simplex virus replication

**DOI:** 10.1186/s12985-016-0470-1

**Published:** 2016-01-27

**Authors:** Carolyn Botting, Xu Lu, Steven J. Triezenberg

**Affiliations:** Van Andel Research Institute, 333 Bostwick Ave NE, Grand Rapids, MI 49503 USA; Department of Biology, University of Findlay, 1000 N Main St, Findlay, OH 45840 USA

**Keywords:** Herpes simplex virus, DNA damage, H2AX, γH2AX, Ataxia telangiectasia mutated, ATM, ATR, Human foreskin fibroblast

## Abstract

**Background:**

Herpes simplex virus type 1 (HSV-1) can establish both lytic and latent infections in humans. The phosphorylation of histone H2AX, a common marker of DNA damage, during lytic infection by HSV-1 is well established. However, the role(s) of H2AX phosphorylation in lytic infection remain unclear.

**Methods:**

Following infection of human foreskin fibroblasts by HSV-1 or HSV-2, we assayed the phosphorylation of H2AX in the presence of inhibitors of transcription, translation, or viral DNA replication, or in the presence of inhibitors of ATM and ATR kinases (KU-55933 and VE-821, respectively). We also assayed viral replication in fibroblasts in the presence of the kinase inhibitors or siRNAs specific for ATM and ATR, as well as in cell lines deficient for either ATR or ATM.

**Results:**

The expression of viral immediate-early and early proteins (including the viral DNA polymerase), but not viral DNA replication or late protein expression, were required for H2AX phosphorylation following HSV-1 infection. Inhibition of ATM kinase activity prevented HSV-stimulated H2AX phosphorylation but had only a minor effect on DNA replication and virus yield in HFF cells. These results differ from previous reports of a dramatic reduction in viral yield following chemical inhibition of ATM in oral keratinocytes or following infection of ATM^−/−^ cells. Inhibition of the closely related kinase ATR (whether by chemical inhibitor or siRNA disruption) had no effect on H2AX phosphorylation and reduced viral DNA replication only moderately. During infection by HSV-2, H2AX phosphorylation was similarly dispensable but was dependent on both ATM activity and viral DNA replication.

**Conclusion:**

H2AX phosphorylation represents a cell type-specific and virus type-specific host response to HSV infection with little impact on viral infection.

**Electronic supplementary material:**

The online version of this article (doi:10.1186/s12985-016-0470-1) contains supplementary material, which is available to authorized users.

## Background

Herpes simplex virus type 1 (HSV-1) has a double-stranded DNA genome of approximately 152 kilobasepairs. Like other herpes viruses, HSV-1 infection is characterized by two distinct cycles; a productive lytic infection and a nonproductive latent infection. During lytic infection, viral genes are expressed in a cascade of at least three classes, commonly designated the immediate-early (IE), early (E), and late (L) genes. Proteins encoded by IE genes facilitate expression of E and L genes. The E genes primarily encode the viral DNA replication machinery. L genes, which are sometimes separated into leaky late and true late genes, encode proteins involved in the structure and assembly of the virus particle. Viral DNA replication is required to express true late genes and enhances leaky late gene expression [1, 2].

The interplay between viral and cellular processes during HSV replication is complex. The virus can subvert cellular mechanisms to enhance the infection process; in response, the cell has many antiviral mechanisms in place to protect its functions. Histone variant H2AX is one example of the complex nature of these virus-host interactions. H2AX constitutes about 10 % of the total H2A distributed through the chromatin of a typical cell. The particular functions of H2AX are still poorly understood, but H2AX is phosphorylated in response to DNA damage at sites up to several kilobasepairs around the site of damage [3]. Disruption of the mouse *H2afx* gene encoding H2AX results in genomic instability and hypersensitivity to radiation [4, 5]. In HSV-1 infection, H2AX is phosphorylated during viral E gene expression, and the amount of phosphorylated H2AX (γH2AX) increases as the gene cascade continues [6–8]. This post-translational modification could reflect host responses attempting to limit the infection process; it could be beneficial to the virus; or it could be a host response without meaningful consequences for viral infection.

Incoming linear HSV viral genomes inherently have free DNA ends that conceivably might initiate a cellular DNA damage response [9]. But the mere delivery of viral DNA into the cell is likely insufficient to trigger H2AX phosphorylation, because that phosphorylation occurs well after viral entry [6]. An alternative hypothesis predicts that the replication or recombination of the viral DNA bearing single-strand nicks and gaps will initiate a DNA damage response including H2AX phosphorylation. To date, the mechanisms of H2AX phosphorylation during HSV infection and the effects on viral replication remain incompletely defined.

H2AX is a direct substrate for phosphorylation by the host cell kinases ATM (ataxia telangiectasia mutated) and ATR (ataxia telangiectasia and Rad3-related), which along with DNA-PK are the central signaling proteins of the DNA damage response pathway. ATM and DNA-PK typically respond to double-strand breaks, whereas ATR responds to single-strand DNA and stalled replication forks [10]. The potential roles of these protein kinases in HSV infection have been investigated [7, 11–17]. Others have shown that the viral IE protein ICP0 induces proteasome-mediated degradation of the catalytic subunit of DNA-PK and that the loss of DNA-PK activity increases virus replication [12, 18]. The kinase function of ATM is activated during viral DNA replication [11, 14, 15], and reduced HSV-1 replication in ATM-deficient cell lines suggests that ATM is important for viral replication during lytic infection [11]. Li et al. [6] and Alekseev et al. [19] also found that an inhibitor specific for ATM (KU-55933) resulted in a decrease of HSV-1 at low multiplicity of infection (MOI) in AD-293 and OKF9 cells, respectively. In contrast, Shirata et al. [14] reported that knockdown of ATM had no effect on HSV-2 infection in 293 T cells. This difference in ATM dependence between HSV-1 and HSV-2 is curious. In addition, we do not yet know the trigger responsible for H2AX phosphorylation during HSV infection nor, more importantly, whether γH2AX plays an active role in production of HSV.

We report here that ATM activity (but not ATR activity) and the expression of viral proteins (including UL30, the viral DNA polymerase), but not viral DNA replication per se, are necessary for HSV-1-induced H2AX phosphorylation in human foreskin fibroblasts. Intriguingly, during infection of fibroblasts by HSV-2, H2AX phosphorylation does require viral DNA replication. However, reducing H2AX phosphorylation by chemical or siRNA inhibition of ATM did not significantly affect HSV-1 or HSV-2 DNA replication and virus production at high MOI, and had only a modest effect at lower MOI. These results differ from reports [6, 19] which suggest that ATM performs an important role in HSV-1 infection of other cell lines. Collectively, these observations suggest that H2AX phosphorylation represents a cell-specific and virus-specific host response to HSV infection and that such phosphorylation has little impact on viral infection.

## Methods

### Cells and viruses

Vero cells were obtained from ATCC and telomerase-transformed human foreskin fibroblasts (HFFs) were provided by Wade Bresnahan [20]. Vero and HFF cells were maintained in Dulbecco's modified Eagle's medium (DMEM) supplemented with 10 % fetal bovine serum (FBS). Vero-PolB3 cells [21] a gift from Dr. Don Coen, are stably transformed with the viral UL30 gene and express the viral DNA polymerase from its native promoter. These cells were maintained in DMEM with 10 % FBS and 400 μg/ml G418. The primary human skin fibroblast cell lines GM01588A and GM02052F (ATM^−/−^ cell lines each with a point mutation resulting in early termination of ATM [22]), GM18366D (expressing low levels of ATR due to a point mutation resulting in alternate splicing [23]), and GM05757B (expressing wild-type ATM and ATR) were obtained from Coriell Institute (Camden, NJ). GM01588A, GM02052F, and GM05757B cells were maintained in DMEM plus 10 % FBS; GM18366D cells were maintained in DMEM plus 20 % FBS. HSV-1 virus strain KOS and HSV-2 virus strain G were propagated and titered in Vero cells. HSV-1 strains HP66 and ΔS1, both bearing deletions of the viral DNA polymerase gene UL30, were obtained from Dr. Coen and were grown and titered in Vero-PolB3 cells [21].

### Reagents

Antibodies against H2AX (ABCM-AB10475) and phosphorylated H2AX (γH2AX) (ABCM-AB4178) were purchased from Abcam. UL30 antibody was a gift from Dr. Nigel Stow [24]. GAPDH and actin antibodies were obtained from Millipore (MAB374 and MAB1501). The siRNAs were purchased from QIAGEN, including control siRNAs (SI03650318 and SI04381048) and validated siRNAs directed against ATM (ATM1 = SI00299299 and ATM2 = SI00604730) or ATR (ATR1 = SI02660231 and ATR2 = SI02664347). A third siRNA directed against ATR, here designated ATR3, is identical in sequence to an shRNA used by Dr. Sandra Weller and colleagues [13, 17]. Transfections were done with SiLentFect (BioRad). Kinase inhibitors KU-55933 [25] and CGK733 [26–29] were purchased from EMD (118500 and 118501). VE-821 [30] was purchased from AdooQ Bioscience (A11605). Actinomycin D (A9415), cycloheximide (C7698), phosphonoacetic acid (PAA) (284270) and doxorubicin (D1515) were purchased from Sigma. The 2X PhosphoStop phosphatase inhibitor cocktail and 2X minicomplete protease inhibitor cocktail (04906845001 and 11836153001) were both purchased from Roche. TRIzol (15596–026) was purchased from Invitrogen. Reverse transcription reactions were performed with a High Capacity cDNA Reverse Transcription Kit (ABI). SYBR Green master mix (04673522001) was purchased from Roche. CellTiter-Glo viability kit was purchased from Promega (G7570).

### Gene expression and quantitative PCR

Total RNA was purified from cells using TRIzol reagent or the Qiagen RNeasy kit and was reverse-transcribed with random primers and RNAseOut according to the manufacturer’s protocol. Gene expression was quantified by real-time quantitative PCR (qPCR) using primers specific for the selected viral or human genes. DNA samples were collected using Qiagen DNeasy Blood and Tissue Kit and were quantified by qPCR with primers specific for viral ICP4 or ICP0 genes or the human 18S ribosomal RNA gene. The qPCR was performed on an ABI 7500 RT-PCR system (Applied Biosystems) using SYBR Green master mix; relative DNA or RNA levels were analyzed by the 2^–ΔΔCt^ method.

### HSV infection

HSV-1 and HSV-2 infections in HFF cells were performed as follows. Cells were washed with DMEM, inoculated with the appropriate MOI, and incubated at 37 °C. At 1 h post-infection (h p.i.) the inoculum was aspirated and cells were washed with DMEM. Cells were incubated in DMEM containing 10 % serum at 37 °C and were harvested at various times. In kinase inhibitor experiments, KU-55933, VE-821, or CGK733 (or DMSO vehicle) was added to cell culture wells at 1 h p.i. in normal media. For inhibition of viral DNA replication, PAA was added at 1 h p.i. to the normal medium to a final concentration of 400 μg/ml. For infections using actinomycin D (1 μg/ml) or cycloheximide (100 μg/ml), the inhibitors were added to the cells for 1 h before infection and were present during the entire course of infection.

Infections for plaque assays were performed as described above, except that DMEM with 2 % FBS and 0.9 % SeaPlaque agarose was added to the infected cells at 1 h p.i. At 3 d p.i. the cells were stained with neutral red for 2 h at 37 °C to visualize plaques.

### Western blot

Lysates analyzed by western blot were collected as follows. After the cell culture medium was aspirated, the cells were washed with phosphate-buffered saline (PBS) and were lysed directly in 2X SDS-PAGE loading buffer containing 2X PhosphoStop phophatase inhibitor cocktail and 2X minicomplete protease inhibitor cocktail with 5 % β-mercaptoethanol. The lysates were heated at 95 °F for 5 min and separated on 4-20 % polyacrylamide gels. The proteins were transferred to polyvinylidene fluoride membranes and blocked in 1X TTBS (100 mM Tris, pH 7.5, 150 mM NaCl, 0.1 % Tween 20) containing 2 % or 5 % (w/v) bovine serum albumin. The blocked membrane was incubated with primary antibody overnight at 4 °C at the dilution suggested by the supplier. The membrane was washed with TTBS and incubated with the appropriate secondary antibody conjugated with horseradish peroxidase at 5000-fold dilution for 1 h at room temperature. The membrane was again washed and the horseradish peroxidase was detected by chemiluminescence.

### siRNA transfections

siRNA transfections of HFF cells were performed using the transfection reagent SilentFect according to the manufacturer’s protocol, with a final siRNA concentration of 16.7 nM. At 24 h post-transfection, the transfection mixture was aspirated and replaced with medium containing DMEM plus 10 % FBS. Cells were incubated at 37 °C for approximately 72 h post-transfection. Cells either were harvested for analysis of the knockdown efficiency or were infected with HSV using the infection protocol described above. Total RNA and DNA were collected at 16 h p.i. and the amount of viral RNA or DNA was measured by qPCR and normalized to host 18S DNA and negative control siRNA samples.

### Nuclei extraction by gradient centrifugation

ATR-deficient and HFF cells were infected as detailed above. At 2 h p.i., cells were trypsinized, resuspended in 10 % FBS DMEM, and counted to ensure equal numbers of cells were loaded into each gradient. Gradients were prepared and centrifuged as described previously [31], except that Optiprep media (Sigma) was used as the gradient material and dilutions were modified accordingly to obtain the correct percentage of iodixanol for each layer. Nuclear and cytoplasmic fractions were harvested and tested for purity by qPCR with GAPDH and 18S primers, as well as by immunoblotting with GAPDH and histone 3.3 antibodies. DNA in infected samples was quantified by qPCR with ICP0 and 18S rRNA primers.

## Results

### H2AX phosphorylation during infection by HSV-2 (but not HSV-1) requires viral DNA synthesis

**Fig. 1:**
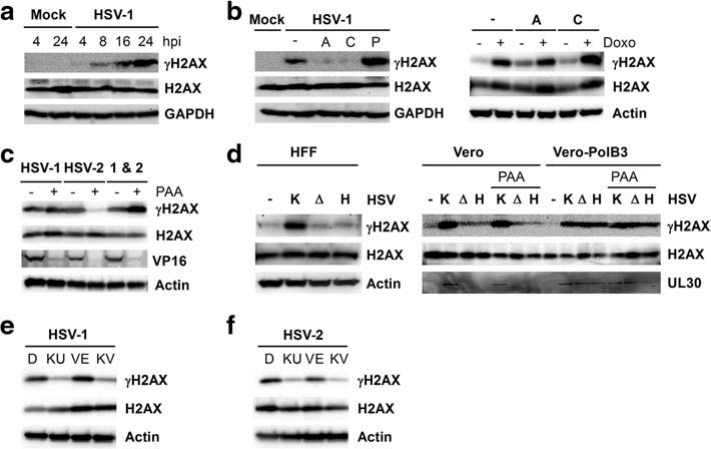
HSV-induced H2AX phosphorylation requires the presence of the viral polymerase and ATM kinase activity. **a** Human foreskin fibroblast (HFF) cells were infected with HSV-1 (MOI 5) and cell lysates were prepared at various times post-infection. The lysates were immunoblotted and probed with antibodies for total H2AX or its phosphorylated counterpart (γH2AX), with GAPDH as a loading control. **b** The transcription inhibitor actinomycin D (A, 1 μg/ml) and the translation inhibitor cycloheximide (C, 100 μg/ml) were added to HFFs 1 h before HSV-1 infection or 1 μM doxorubicin (Doxo) treatment. The DNA synthesis inhibitor phosphonoacetic acid (P, 400 μg/ml) was added at 1 h p.i. All compounds remained in the media until lysis at 24 h p.i. **c** HFFs were infected with HSV-1 and/or HSV-2 with or without phosphonoacetic acid (PAA, 400 μg/ml). Cells were lysed at 24 h p.i., immunoblotted, and probed for total H2AX, γH2AX, or VP16, with actin loading control. **d** Vero or HFF cells were either mock-infected, infected with HSV-1 KOS strain (K), or infected with a mutant virus lacking UL30, either strain ΔS1 (Δ) or HP66 (H) at MOI 5. Cell lysates were prepared at 16 h.p.i. and analyzed by immunoblot. **e** and **f** HFFs were infected with HSV-1 (panel **e**) or HSV-2 (panel **f**), immediately overlaid with an ATM inhibitor (KU-55933, KU), an ATR inhibitor (VE-821, VE), or the two in combination (KV), and lysed at 24 h p.i. prior to immunoblot analysis

A number of viruses including HSV induce the host DNA damage response pathway and subsequent phosphorylation of histone H2AX [6, 7]. However, the mechanism that triggers this pathway and its importance for viral replication during HSV infection still remains unclear. To explore this, we first verified that HSV-1 was sufficient to initiate γH2AX formation in telomerase-transformed human foreskin fibroblasts (HFF). HFF cells were infected with HSV-1 at high multiplicity (MOI 5), and lysates from several time points post-infection were collected for immunoblotting using an antibody that detects γH2AX. γH2AX was faintly detectable at 4 h post-infection (h p.i.) and levels of γH2AX increased during the course of infection (Fig. 1a). This pattern of γH2AX induction well after the start of infection indicates that the phosphorylation is unlikely to be induced simply by entry of virion DNA.

To more precisely determine the requirements for inducing γH2AX, we used actinomycin D to interrupt mRNA synthesis, cycloheximide to inhibit translation, or phosphonoacetic acid (PAA) to disrupt viral DNA replication. HFF cells infected with HSV-1 in the presence of either actinomycin D or cycloheximide showed inhibition of H2AX phosphorylation (Fig. 1b). We conclude that *de novo* synthesis of one or more proteins in the infected cell is required to trigger H2AX phosphorylation. As a control, we tested whether *de novo* protein synthesis is always necessary for ATM-mediated H2AX phosphorylation. When doxorubicin was used to induce double-stranded DNA breaks, neither actinomycin D nor cycloheximide inhibited γH2AX formation (Fig. 1b, right panel), indicating that transcription and translation are not inherently required for H2AX phosphorylation in fibroblasts. Additionally, although the presence of PAA during a HSV-1 infection potently inhibited viral DNA replication and thus late protein (VP16) synthesis (Fig. 1c), the presence of PAA did not inhibit H2AX phosphorylation during HSV-1 infection (Fig. 1b and c). This result indicates that neither viral DNA replication nor true late proteins are required for induction of γH2AX in HSV-1 infection.

Curiously, however, PAA did inhibit H2AX phosphorylation induced upon infection by HSV-2 (Fig. 1c). Thus, DNA synthesis (and perhaps late protein expression) is required to trigger γH2AX during infection by HSV-2 but not by HSV-1, an unexpected difference between these two closely related viruses. Furthermore, co-infection by HSV-1 and HSV-2 resulted in robust levels of γH2AX that were not diminished by PAA, revealing that HSV-2 infection does not elicit active repression of H2AX phosphorylation. These observations indicate distinct differences in the DNA damage response pathway involvement during HSV-1 and HSV-2 infection, despite the many molecular similarities between the two viruses.

### UL30 is required for H2AX phosphorylation during HSV-1 infection

The observation that addition of PAA did not diminish H2AX phosphorylation during HSV-1 infection suggested that viral DNA synthesis is not necessary for this manifestation of the DNA damage response. As a further test of this hypothesis, we predicted that infection by HSV-1 mutants defective in viral DNA replication would nonetheless induce γH2AX. We used two HSV-1 mutant strains, HP66 and ΔS1 [21], which have substantial deletions in the UL30 gene (encoding the viral DNA polymerase), to infect complementing Vero-PolB3 [21] or non-complementing Vero or HFF cells at MOI 5. Surprisingly, neither HP66 nor ΔS1 was able to induce significant phosphorylation of H2AX in Vero or HFF cells (Fig. 1d). However, the HP66 or ΔS1 strains did induce γH2AX formation in the Vero-PolB3 cells (which express UL30 upon infection) regardless of PAA addition (Fig. 1d). We conclude that expression of the wild-type viral DNA polymerase protein, but not active viral DNA synthesis, is required to induce H2AX phosphorylation in cells infected by HSV-1.

### ATM (but not ATR) is required for HSV H2AX phosphorylation

To interrogate the role of phosphorylated H2AX in infection of human fibroblasts by HSV-1 and HSV-2, we inhibited ATM and ATR activity using three approaches: chemical inhibitors of ATM or ATR kinase activity, siRNAs specific for ATM and ATR, and cell lines deficient in either ATR or ATM.

To determine whether ATM, ATR, or both are required for H2AX phosphorylation, we used two small-molecule kinase inhibitors. KU-55933 inhibits ATM activity [25], whereas VE-821 inhibits ATR activity [30, 32]. We infected HFFs with HSV-1 or HSV-2 (MOI 5), applied the kinase inhibitors, and harvested the cells at 24 h p.i. As shown in Fig. 1e and f, KU-55933 repressed H2AX phosphorylation in cells infected by either HSV-1 or HSV-2, but VE-821 did not, indicating that ATM, not ATR, was responsible for H2AX phosphorylation.

We also performed comparable experiments with CGK733, which reportedly inhibits both ATM and ATR activity. Although the original report identifying CGK733 as an ATM and ATR inhibitor [29] was retracted [33], the inhibitory activity of CGK733 has since been verified by independent laboratories [26–28]. CGK733 repressed γH2AX in cells infected by HSV-2 but, to our surprise, not in cells infected by HSV-1 (data not shown). However, at the concentration of CGK733 required for repression of γH2AX (10 μM), the drug also showed substantial cytotoxicity as assessed by CellTiter-Glo reagent after 24 h (CC_50_ 5.3 ± 1 μM) and a strong caspase-3/7 response suggestive of apoptosis (ApoTox-Glo assay) (data not shown). Although the differential effect of CGK733 on HSV-1 and HSV-2 infection is intriguing, CGK733 was not used further because of the cytotoxicity in HFF cells.

### Neither ATM nor ATR activity is required for HSV-1 DNA replication

**Fig. 2:**
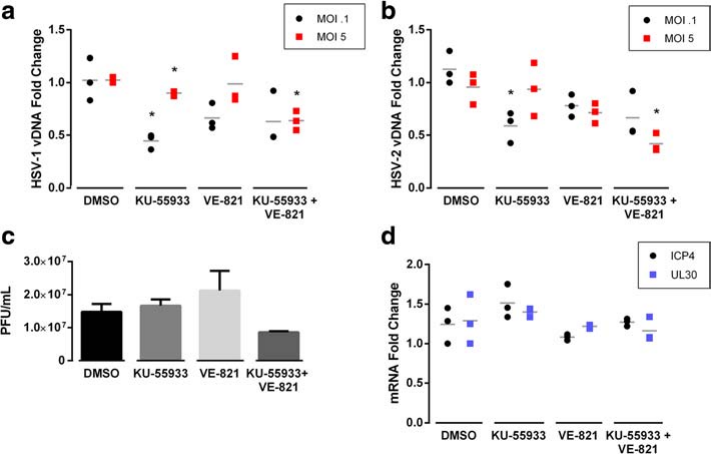
ATM and ATR inhibitors moderately reduce HSV-1 DNA replication and virion production. **a** and **b** HFFs were infected with HSV-1 (panel **a**) or HSV-2 (panel **b**) and immediately overlaid with ATM (KU-55933) or ATR (VE-821) kinase inhibitors alone or in combination. Total DNA was harvested at 24 h p.i. and viral DNA (vDNA) was quantified by qPCR. ICP0 levels were normalized to 18S controls and to DMSO-treated samples using the 2^–ΔΔCt^ method. Grey lines indicate the average of three experiments with infections at either low MOI (0.1; circles) or high MOI (5; squares). **c** Plaque assays were performed on a representative experiment from panel (**a**). Each column represents the average and standard deviation of viral yield from biological triplicates. **d**. HFFs were infected with HSV-1 at MOI 5 in the absence or presence of kinase inhibitors. Viral gene transcript levels were quantified at 6 h p.i. by qPCR and normalized to 18S and DMSO levels (grey line denotes average of three experiments). Asterisks indicate results that are statistically significant relative to DMSO control at *p* < 0.05

Having established that HSV-induced H2AX phosphorylation depends on ATM but not on ATR, we sought to determine if inhibiting this phosphorylation impacts HSV-1 and HSV-2 viral production. We measured the amount of viral DNA in infected cells in the presence of the ATM and ATR inhibitors, either alone or in combination. The relative concentrations of host DNA (18S rRNA gene) and viral DNA (ICP0 gene) were measured by qPCR and analyzed using the 2^–ΔΔCt^ method. As shown in Fig. 2a and b, KU-55933 moderately reduced viral DNA production of both viruses, with slightly more effect at low MOI. VE-821 had little effect on replication regardless of virus or MOI, and the two compounds together did not reduce replication further than KU-55933 alone. Under these experimental conditions, KU-55933 repressed H2AX phosphorylation, but VE-821 did not (see Fig. 1e and f). We conclude that neither ATM and ATR activity, nor H2AX phosphorylation, is required for viral DNA production in HFF cells.

### ATM and ATR are dispensable for virus production

Although KU-55933 and VE-821 had little effect on viral DNA replication, whether the inhibitors might have an effect on other steps of virus production remained unanswered. To address this, supernatants were harvested from a representative experiment in Fig. 2a and infectious virus yield was quantified by plaque assay (Fig. 2c). Neither KU-55933 nor VE-821 alone diminished viral yield and the two inhibitors in combination reduced viral yield by less than twofold. Furthermore, neither compound alone nor the combination affected either IE gene (ICP4) or E gene (UL30) expression as demonstrated by RT-qPCR assays (Fig. 2d). These data indicate that both ATM and ATR are ultimately dispensable for all steps of HSV virus production in HFF cells.

### siRNAs confirm that ATM and ATR are not required for HSV infection

**Fig. 3:**
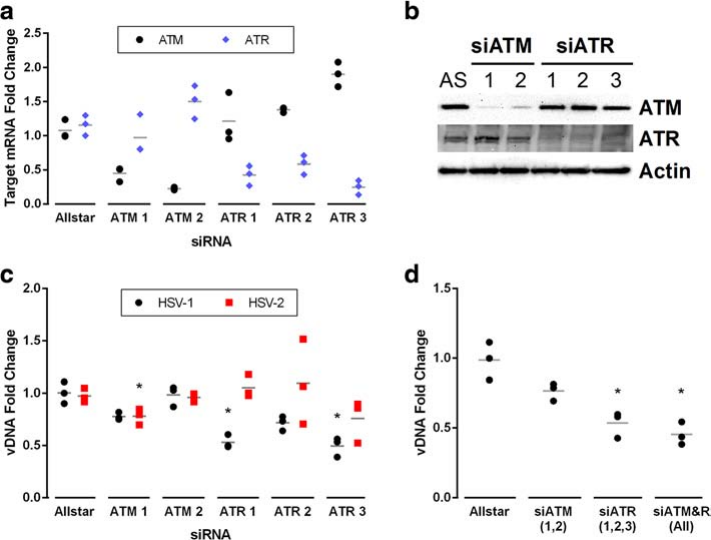
ATM and ATR siRNAs moderately reduce viral DNA production. HFFs were transfected with siRNAs targeting ATM or ATR. At 72 h post-transfection, cells were infected with HSV-1 or HSV-2 (MOI 5) and harvested at 16 h p.i. **a** ATM and ATR transcript levels in siRNA-transfected cells were quantified using qPCR, normalized to 18S and control siRNA (Allstar, AS) by the 2^–ΔΔCt^ method. **b** Protein lysates of siRNA-transfected cells were immunoblotted and probed with antibodies specific for ATM or ATR. **c** Viral DNA levels (vDNA) were quantified by qPCR with ICP0 primers, normalized to 18S rDNA levels and Allstar control siRNA. **d** HSV-1 DNA levels in HFFs transfected with pooled ATM and/or ATR siRNAs were quantified by qPCR. In panels (**a**), (**c**), and (**d**), grey lines indicate the average of three experiments. Asterisks indicate statistical significance relative to Allstar control at *p* < 0.05

To further confirm that neither ATM nor ATR are required, HFF cells were transfected with siRNAs against ATM or ATR, either alone or in combination, and were then infected with HSV-1 or HSV-2 (MOI 5) approximately 72 h post-transfection. Total RNA and DNA were harvested at 16 h p.i. and viral transcripts or genomes were quantified with qPCR. The efficacy and specificity of the siRNAs was evident in reduced transcript and protein amounts for the targeted kinases (Fig. 3a and b). However, disruption of either ATM or ATR expression had little effect on HSV-1 or HSV-2 DNA replication (Fig. 3c), further indicating that neither kinase is vital for viral replication. When several siRNAs targeting ATM and ATR were combined, we observed a modest reduction of HSV-1 viral DNA (Fig. 3d), similar to that of the chemical inhibitors. These data further confirm that ATM and ATR activity, and thus H2AX phosphorylation, are not required for HSV replication.

### HSV-1 DNA replication is greatly reduced or delayed in ATR- or ATM-deficient cells

**Fig. 4:**
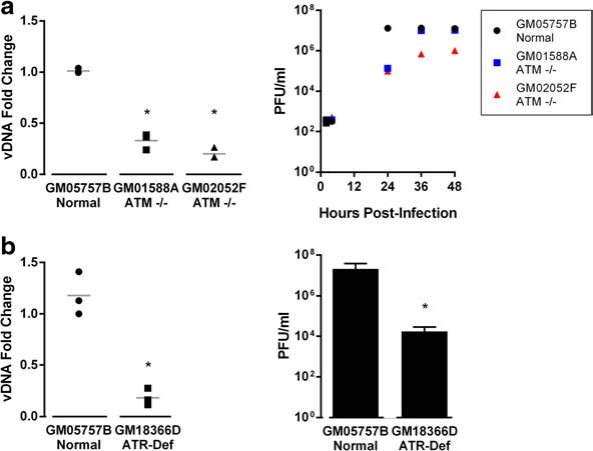
HSV-1 replication and virion production are reduced in both ATM^−/−^ and ATR-deficient cell lines. ATM^−/−^ cells (GM01588A and GM02052F), ATR-deficient (GM18366D) cells, or normal human fibroblasts (GM05757B) were infected with HSV-1 (MOI 5). **a**. Infection of ATM^−/−^ cells. **b**. Infection of ATR-deficient cells. Left panels: At 24 h p.i., total DNA was harvested from cells and supernatant. Viral DNA (vDNA) was quantified using ICP0 primers and normalized to 18S rDNA and GM05757B values. Grey lines indicate mean of triplicate experiments. Right panels: Plaque assays were performed using supernatants collected at various times from infected cells. Bars represent the average and standard deviation of biological triplicates. Asterisks indicate statistical significance relative to wildtype fibroblasts at *p* < 0.05

A previous report indicated that HSV-1 requires ATM for efficient replication based on evidence of reduced viral replication in ATM^−/−^ cells [11]. Since the data described thus far in this report contradict that earlier conclusion, we decided to perform comparable experiments. Two independent ATM^−/−^ cell lines (GM01588A and GM02052F, each expressing no ATM due to an early stop codon) and one ATR-deficient cell line (Seckel syndrome, GM18366D, expressing low levels of ATR due to alternate splicing), as well as a wildtype fibroblast line (GM05757B), were procured from the Coriell Institute. Cells from each of these lines were infected with HSV-1 to compare the production of viral DNA and infectious progeny. Both of the ATM^−/−^ cell lines produced much less viral DNA and fewer infectious progeny at 24 h p.i. than did wildtype fibroblasts, as measured by qPCR and plaque assay (Fig. 4a). Both ATM^−/−^ lines produced somewhat higher titers at later times post-infection, (Fig. 4a, right panel), although viral yield from the GM02052F line remained 1 log lower than from wildtype cells. A similar reduction in viral DNA and progeny virus was observed in the ATR-deficient cells at 24 h p.i. (Fig. 4b). We attempted to restore viral replication in ATR-deficient GM18366D cells by transfecting a plasmid expressing wild-type ATR protein, but a strong interferon response to the transfected DNA led to reduced efficiency of infection (data not shown). Thus, we cannot confidently conclude that the poor replication in these cells is due solely to the ATR deficiency. These results seem to suggest that ATM and ATR are important for viral replication, in agreement with the previous report [11] but at odds with our results using kinase inhibitors and siRNAs.

To test whether this reduction of viral DNA replication in ATR-deficient cells was due to inefficient infection or trafficking to the nucleus, we quantified the amount of viral DNA in the nucleus very early in infection. The fibroblast control and ATR-deficient cells were infected with HSV-1 (MOI 5), and at 2 h p.i. the cells were collected. Nuclei were isolated via gradient centrifugation [31] and qPCR was used to quantify viral genomes present in the nuclear fraction. Viral DNA was present at comparable levels in control and deficient nuclei (Additional file 1: Figure S1), indicating the observed viral DNA reduction is not due to attenuated entry of viral DNA to the nucleus. Since these cells likely have adapted mechanisms to overcome the loss of ATM or ATR, it is possible that the altered cellular pathways are not adequate to support efficient HSV-1 replication. Together, these data suggest that the dependence of HSV DNA replication on ATM and ATR is cell-type-specific.

## Discussion

A number of viruses make use of the cellular DNA repair machinery to promote successful infection. One of the first proteins to be activated upon DNA damage is H2AX [34]. Phosphorylated H2AX (γH2AX) spreads over chromatin flanking the DNA damage site [35] and acts as a signal for recruiting other DNA damage response proteins [36]. Others have noted that HSV-1infection is sufficient to induce γH2AX formation [6, 7]. We too observed that HSV-1 infection induced γH2AX formation at 4 h p.i. and that the phosphorylation increased over time. We found that *de novo* protein synthesis is required for H2AX phosphorylation but neither viral replication nor late viral protein expression is required for this phosphorylation event. Thus, induction of H2AX phosphorylation is not triggered merely by the presence of viral DNA in the infected cell nor by replication of viral DNA, but by expression of some viral or cellular protein following infection.

Curiously, although viral DNA replication is not required to induce γH2AX, expression of the viral DNA polymerase is required. Infection by two different viral mutants, HP66 and ΔS1 (each bearing substantial deletions of the UL30 open reading frame), failed to induce γH2AX. It is puzzling that expression of the viral DNA replication enzyme, but not DNA replication itself, is required for the formation of γH2AX during HSV-1 infection. We infer that H2AX phosphorylation is triggered by an event that occurs after UL30 expression but before HSV-1 DNA replication, perhaps during establishment of replication centers. This notion agrees with work by Wilkinson and Weller [7, 15] showing that γH2AX accumulates around the viral replication compartments. Combined with the observation that ATM is solely required for H2AX phosphorylation, we speculate that the viral DNA polymerase could be involved in the recruitment of ATM to replication compartments, although further study would be needed to establish this point.

A second surprising observation was that H2AX phosphorylation during HSV-2 infection was blocked by the viral DNA replication inhibitor PAA, in stark contrast to HSV-1 infection. We presume that expression of UL30 is also required for H2AX phosphorylation during HSV-2 infection because viral DNA synthesis is required, but HSV-2 UL30 mutants are not readily available to test that presumption directly. We therefore infer the DDR pathway is activated differently in HSV-1 and HSV-2 infections; more work will be needed to better understand the mechanistic differences between these two closely related viruses.

Activation of ATM and at least some of its downstream targets by both HSV-1 and HSV-2 has been shown previously [11, 14, 15, 37, 38]. Our results indicate that during HSV-1 or HSV-2 infection, H2AX was phosphorylated solely by ATM. Furthermore, inhibition of ATM has been shown to decrease HSV-1 viral DNA replication in certain contexts [11, 19]. However, in our experiments, chemical inhibition of ATM and/or ATR or disruption of ATM and ATR expression by siRNAs had little or no effect on HSV-1 and HSV-2 DNA replication and virus production. Therefore, although ATM signaling is activated by HSV-1 infection, ATM is not vital for efficient HSV replication in HFF cells, and thus γH2AX formation is likely an incidental signal during infection. Since ATM is activated and γH2AX is formed, it seems reasonable to consider whether ATR is also activated. Others have shown that viral proteins prevent the phosphorylation of typical ATR targets during HSV-1 infection, and shRNA disruption of ATR alone has little effect on viral yield [11, 13–17, 39]. In our hands as well, ATR activity does not contribute substantially to phosphorylation of H2AX during HSV infection, and ATR activity is not required for effective viral replication. Moreover, HSV-1 was insufficient to activate Chk-1 and thus downstream ATR activation (data not shown).

ATM and the γH2AX response have also been studied in the context of infection by other herpesviruses. Conflicting reports suggest that ATM is required for maximum yield of human cytomegalovirus and for maturation of replication compartments in HEL fibroblasts [40], yet ATM^−^ cells had only a 1 log reduction in viral yield as compared to HFF cells [41]. The literature is likewise divided over the role of ATM kinase activity during EBV infection; one report [42] concludes that ATM is important for efficient EBV lytic replication in Akata BX1 latent cells but another report [43] indicates that ATM is dispensable for EBV lytic replication in Tet-BZLF1/B95-8 cells. A comprehensive report of various stimuli of EBV in various cell lines indicates that strict dependence on ATM is stimulus-specific; ATM was vital for early steps in reactivation but not for viral DNA replication per se [44]. Furthermore, murine herpesvirus 68 required ATM and γH2AX in primary mouse macrophages only at low MOI, but neither are required for growth in MEFs regardless of MOI [45, 46]. Karposi sarcoma virus depends on ATM, as shown by reduced establishment and latency of KSHV in the absence of ATM [47, 48]. However, VZV does not seem to depend similarly on ATM since it displayed near normal production in GM02530 ATM^−^ cells [49]. The varying reports for CMV, EBV and HV68 together with the data in this report indicate that cell type is a key factor in virus reliance on DNA damage pathways during herpes virus infection.

In contrast with our results using chemical inhibitors and siRNAs targeting ATM and ATR, but consistent with reports from others [11, 19], we observed a reduction of both viral DNA replication and viral yield upon infecting either ATM^−/−^ cell lines or an ATR-deficient cell line at 24 h. A longer timecourse in the ATM^−^ cells did reveal higher yield of virus at later times, with one line eventually reaching levels comparable to wildtype cells. Our observations support those of Lilley et al. [11], who observed that the yields of HSV-1 from three ataxia telangiectasia lines were reduced by two orders of magnitude at 24 h p.i., although two lines did recover to near wt yield at 36 h p.i. In contrast, Yamamoto et al. [49] showed that both VZV and HSV-2 produced near-normal titers throughout the entire 36 h of infection in GM02530 cells. This discrepancy is echoed by Zavala et al. [50] in their investigation of disparate HCMV production in mutant cells. Although they observed that distinct strains of HCMV yielded different titers in individual ATM^−^ cell lines, they determined that ATM was not necessary for normal titers in fibroblasts. This aligns well with the data we present here for HSV-1. We surmise that the ATM^-^ and ATR-deficient cells may have developed mechanisms to compensate for the lack of DNA damage kinases that are not compatible with efficient HSV-1 replication. Clearly the two ATM^−^ cell lines tested here have adapted in different ways, since they differed in virus production at late times in infection despite having identical mutations in the ATM gene. It is conceivable that the non-homologous end joining pathway, known to be antiviral [18], is more active in these cells leading to decreased or delayed replication. Additionally, fibroblasts and keratinocytes have different efficiencies in supporting replication of UV-damaged HSV, indicating that DDR pathway activation varies significantly between cell types [51]. Inhibition of ATM, and perhaps even of ATR, may be more severe in oral keratinocytes than HFFs because their DNA damage pathways mobilize differently in response to HSV.

The HSV-1 genome is known to contain nicks and single-strand gaps of various sizes that affect infectivity [52]. Moreover, complex replication intermediates have been observed indicative of recombination events [53]. Additionally, the linear genome may circularize prior to lytic replication, though recent reports have differed on this point [54, 55]. Given the role of γH2AX and ATM in homologous recombination, it is logical to hypothesize that these proteins participate in such recombination events during HSV-1 and HSV-2 infection. However, since ATM is dispensable for infection of HFFs, the viral DNA must recombine by means other than canonical ATM-dependent homologous recombination, perhaps through proteins that can complement ATM’s function. The additional disparity between HSV-1 and HSV-2 in γH2AX induction suggests differences in recombination mechanisms during infections by these two closely related viruses. Most DNA replication studies have been done using HSV-1 and inferred for HSV-2, but perhaps these viruses replicate more differently than previously supposed. It would be interesting to determine the circularization and recombination rate of both HSV-1 and HSV-2 in the absence of cellular DDR proteins, especially ATM.

## Conclusion

In summary, we have shown that H2AX phosphorylation is performed by ATM during HSV-1 and HSV-2 infection. This event during HSV-1 infection is dependent on the presence of the viral DNA polymerase (but not viral DNA synthesis), but in HSV-2 infection it is replication-dependent. Furthermore, although ATM is responsible for infection-induced H2AX phosphorylation, neither ATM nor the closely related ATR kinase is strictly required for efficient HSV production. HSV-1 and HSV-2 replication is known to require recombination events that result in nicked and gapped products. It is likely that this DNA damage signal is a natural cellular response to early steps of HSV replication and is responsible for recruiting ATM and ATR to replication compartments but does not enhance or lessen replication in HFF cells. We conclude that γH2AX is a chromatin mark indicative of a cell-type-specific host response that is not essential for HSV-1 or HSV-2 infection.

## Additional file

Additional file 1: Figure S1. HSV-1 DNA enters nuclei of ATR-deficient cells at comparable levels to that of WT fibroblasts. WT fibroblasts (GM05757B) or AT-deficient fibroblasts (GM18366D) were infected with HSV-1 (MOI 5) and trypsinized at 2 h p.i. Nuclei were isolated via gradient centrifugation and viral DNA was quantified with qPCR. ATR-deficient cells were compared to WT fibroblasts using the 2^–ΔΔCt^ method. Grey bars represent the average of three experiments. (PDF 65 kb)

## Abbreviations

ATM: ataxia telangiectasia mutated
ATR: ataxia telangiectasia and Rad3-related
DDR: DNA damage response
DMEM: Dulbecco’s modified essential medium
FBS: fetal bovine serum
HFF: telomerase-transformed human foreskin fibroblasts
HSV-2: HSV-2, herpes simplex virus type 1 and type 2
E, L: E, L, immediate-early, early, late viral genes
MOI: multiplicity of infection
p.i.: post infection
PAA: phosphonoacetic acid
PBS: phosphate-buffered saline
PFU: plaque-forming units
qPCR: quantitative polymerase chain reaction
RT-PCR: reverse-transcription followed by polymerase chain reaction
siRNA: short interfering RNA
TTBS: Tween-containing Tris-buffered saline
γH2AX: phosphorylated H2AX
