# Supplementary material for: H2AX phosphorylation and DNA damage kinase activity are dispensable for herpes simplex virus replication
Source: Virol J. 2016 Jan 27;13:15. doi: 10.1186/s12985-016-0470-1 (PMC4728825; doi:10.1186/s12985-016-0470-1)
Supplement: Additional file 1: Figure S1. — HSV-1 DNA enters nuclei of ATR-deficient cells at comparable levels to that of WT fibroblasts. WT fibroblasts (GM05757B) or AT-deficient fibroblasts (GM18366D) were infected with HSV-1 (MOI 5) and trypsinized at 2 h p.i. Nuclei were isolated via gradient centrifugation and viral DNA was quantified with qPCR. ATR-deficient cells were compared to WT fibroblasts using the 2–ΔΔCt method. Grey bars represent the average of three experiments. (PDF 65 kb) [file 12985_2016_470_MOESM1_ESM.pdf]

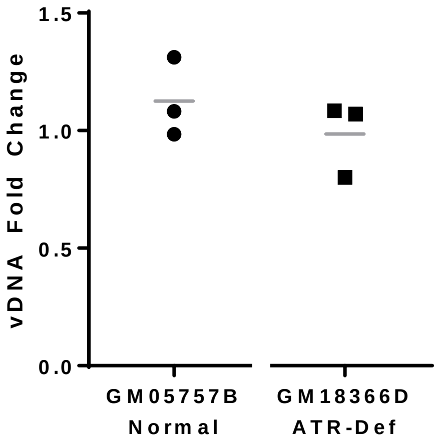

Supplemental Figure: HSV-1 DNA enters ATR-deficient nuclei at comparable levels to that of WT fibroblasts. WT fibroblasts (GM05757B) or AT-deficient fibroblasts (GM18366D) were infected with HSV-1 (MOI 5) and trypsinized at 2 h p.i. Nuclei were isolated via gradient centrifugation and viral DNA was quantified with qPCR. ATR-deficient cells were compared to WT fibroblasts using the $2^{-\Delta\Delta Ct}$ method. Grey bars represent the average of three experiments.
